# Relationship between inflammatory markers, metabolic and anthropometric variables in the Caribbean type 2 diabetic patients with and without microvascular complications

**DOI:** 10.1186/1476-9255-3-17

**Published:** 2006-12-22

**Authors:** B Shivananda Nayak, Lesley Roberts

**Affiliations:** 1 Department of Preclinical Sciences, Biochemistry unit, The University of the West Indies, Trinidad; 2 Department of Medicine, Nephrology unit, EWMSC, Trinidad

## Abstract

**Background:**

Serum sialic acid and C reactive protein are the markers for inflammation. The main objective of this study was to determine the sialic acid level in Caribbean type 2 diabetic patients with and without microvascular complications and its relationship with metabolic and anthropometric variables.

**Research design and methods:**

The Caribbean subjects aged 15–60 years with type 2 diabetes were recruited for the study. Fasting venous blood samples were collected from 162 subjects of which 44 were healthy individuals, 44 were of type 2 diabetes, 44 were of type 2 diabetes with nephropathy and 30 were of diabetes with retinopathy. Simultaneously urine samples were also collected from each of the subjects. All the blood samples were processed for lipid profile, glucose, HbA1_C, _C-reactive protein and sialic acid. The urine samples were analysed for sialic acid and microalbumin.

**Results:**

Serum sialic acid concentrations were significantly higher among diabetic subjects (66.0 ± 11.7 mg %) as compared to controls (55.2 ± 8.3 mg %). There was a significantly increasing trend of serum sialic acid with severity of nephropathy (71.6 ± 23.6 mg %) and degree of urinary albumin excretion (794.3 ± 805.9). The diabetic retinopathy patients also demonstrated significantly higher values of serum sialic acid (77.9 ± 29.0) and urine microalbumin (351.1 ± 559.9). Elevated serum sialic acid microalbumin concentrations were associated with cardiovascular risk factors such as hypertension, increased waist to hip ratios. (P < 0.05). Sialic acid had no correlation with CRP or any component of the lipid profile.

**Conclusion:**

The increased serum sialic acid and microalbumin were strongly related to the presence of microvascular complications like diabetic nephropathy and diabetic retinopathy and cardiovascular risk factors like hypertension and waist to hip ratios in Caribbean type-2 diabetic patients. The serum sialic acid may be used as an inflammatory marker and possible indicator of microvascular complications in type-2 diabetic patients.

## Background

Diabetes Mellitus (DM) is a metabolic disorder of multiple etiologies characterized by chronic hyperglycemia with disturbances of carbohydrate, fat and protein metabolism resulting from defects in insulin secretion, insulin action or both. In 1999, it was the second leading cause of death in Trinidad and Tobago with 1306 deaths [1]. According to the World Health Organization, it costs each diabetic an average of 533 US$ per annum in direct cost with respect to health care, which is over 300% the amount a healthy person would have to pay in health care per annum [2].

The complications of Diabetes Mellitus include cardiovascular disease (CVD) and it has been found that CVD is due in part to low grade systemic inflammation [3]. In the Atherosclerosis Risk in Communities (ARIC) study, there was a positive link between systemic inflammation and the development of type 2 diabetes, but this link was seen only in white non smokers. The link was neither seen among African Americans nor among smokers [4]. However, in our previous study we found a positive correlation between inflammatory markers and type 2 diabetes in the Indian population [5].

Sialic acid is a component of cell membranes [6] and elevated levels may indicate excessive cell membrane damage, but more specifically to the cells of vascular tissue. If there is damage to vascular tissue, this leads to ischaemia and this ischaemia is most visible in the smallest blood vessels, including those of the retina of the eyes, kidneys, heart and brain. It is this ischaemia that leads to conditions including, but not limited to retinopathy, nephropathy and neuropathy. In addition, sialic acid can be used as a measurement of the acute phase response because many of the proteins of the immune response are actually glycoproteins and these glycoproteins have sialic acid as the terminal sugar on their oligosaccharide chain [7].

The purpose of this study would be to determine whether in Caribbean type 2 diabetics, there is a higher level of sialic acid, which is a marker of acute phase inflammation. As mentioned before, there have been studies that have suggested that there is a positive correlation, while others suggest it is only within certain groups that there is a positive correlation. It would, therefore, be interesting to see the results within a multiethnic society such as that of Trinidadians (Caribbean subjects). In our earlier studies we found a positive correlation in the Indian population, this would be of even greater significance in the Caribbean population due to our multiethnic population, of which the descendants of East Indian immigrants make up a significant portion. If there is proper assessment of cardiovascular risk among diabetics here in Trinidad, this would in turn allow medical practitioners to better manage their diabetic patients with regards to prevention of complications and improve not only the life expectancy, but the quality of life of these patients.

## Materials and methods

This was a case-control study [8] comparing the concentrations of inflammatory markers and metabolic variables in the Caribbean population Type 2 diabetes with the concentrations of inflammatory markers and metabolic variables in Caribbean population without diabetes. The study includes 162 subjects (male and female) of which 44 were of healthy individuals, 44 patients with type 2 diabetes, 44 patients with type 2 diabetes and nephropathy and 30 diabetics with retinopathy. The Caribbean subjects aged 15–60 years with type 2 diabetes were recruited for the study.

All the subjects were reported in the morning after overnight fast. Standing height and weight were measured. Body Mass Index (BMI) defined as weight in kg/height (meters) squared was calculated and used as an index of obesity. To determine waist to hip ratio, the standardized clinician's tape measure was placed around the widest part of the hips and then placed around the narrowest part of the waist above the belly button. The ratio was determined by dividing the waist measurement by the hip measurement [9]. The blood pressure was measured according to the standard procedure.

The fasting blood and random urine samples were collected. The red top tubes (without any anticoagulant) were used to collect the blood samples for the analysis of sialic acid,, CRP, total cholesterol, triglyceride (TG), low-density lipoprotein (LDL), high-density lipoprotein (HDL), and grey top tubes (with fluoride) were used to collect the sample for glucose estimation. Green top tubes (with heparin) were used to collect the blood sample for HbA1c determination. The Blood and urine samples were kept on ice prior centrifugation. All the serum and urine samples were stored at -20°C. The lipid profile and sugar was done with automated instrument. The HbA1_C, _CRP and urine albumin were analyzed by NycoCard reader (point of care instrument designed for rapid and reliable measurements of microalbumin, C – reactive protein and HbA1c) supplied from JT Rapid diagnostics, Trinidad and Tobago (Axis Shield PoCAS, Norway, the manufacturer). The materials supplied included were quality control, test device, diluents and washing solution.

Serum and urinary sialic acid were measured by a spectrophotometric assay [10]. In this method 0.15 ml serum (or urine) was mixed with 3.60 ml of 5% TCA and the tubes were covered with marbles, and kept in a boiling water bath for 15 minutes. The tubes were cooled and centrifuged for 10 minutes at 2000 g. Taken 1.0 ml of supernatant and mixed with 2.0 ml each of acid reagent and diphenylamine reagent. Simultaneously standard and blank were treated in the same way. Mix all the tubes, cover with marbles and placed in a boiling water bath for 30 minutes. The purple color produced measured on a spectrophotometer at 540 nm.

### Sample specification

The target population was 15–60 year old Caribbean subjects with type 2 diabetes and sample selection was via stratified random sampling. The inclusion criteria were 15–60 year old Caribbean subjects, both male and female: a) in good overall health, considered as controls, b) with Type 2 diabetes and no complications, c) Type II diabetes with nephropathy. d) Type 2 diabetes with retinopathy. The exclusion criteria were: a) persons with type 1 diabetes, b) persons who had or recently had any medical condition which could affect concentrations of inflammatory markers (for instance, cancer), c) persons taking cholesterol-lowering medication, d) pregnant women and e) heavy smokers (more than one pack of cigarettes per day).

Demographic data collected were: age, gender and ethnicity. Potential confounding variables were, diet, physical activity and obesity.

### Statistical method

Results were expressed as mean ± SD. Data were analyzed using the statistical package for social science (SPSS). The comparisons within and among group were done using one way ANOVA test. The p value < 0.05 was taken as the cut off level for significance. Because the distribution of most variables was not symmetric. Pearson's correlation test was used for correlation study.

## Results

**Figure 1:**
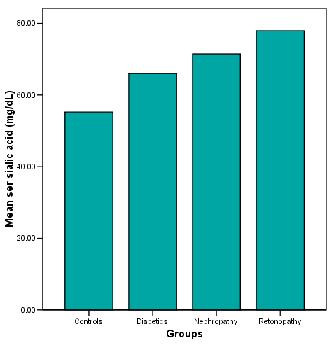
Serum sialic acid concentration in controls (healthy individuals), diabetics, diabetics with nephropathy and diabetics with retinopathy.

**Figure 2:**
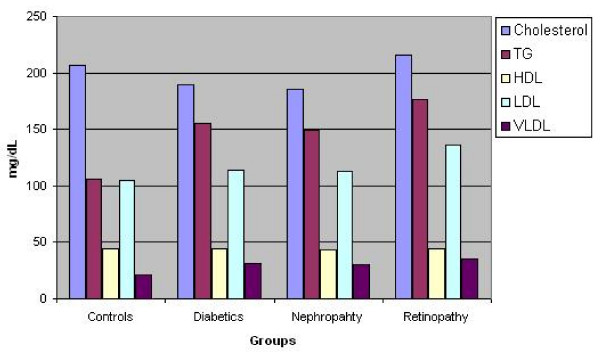
Lipid profile status among the four study groups.

**Figure 3:**
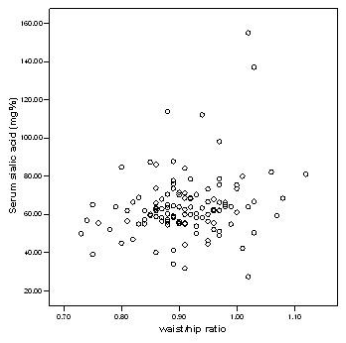
Scatter plot showing the relationship between serum sialic acid concentration and waist to hip ratio.

**Figure 4:**
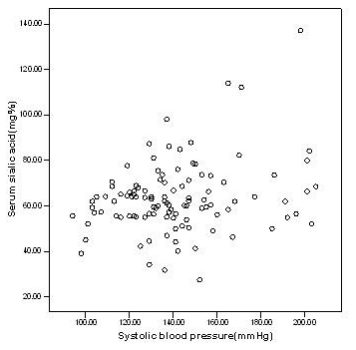
Scatter plot showing relationship between sialic acid and systolic blood pressure mmHg.

Table 1 shows the relationship between inflammatory markers, metabolic and anthropometric variables in Caribbean population with type 2 diabetes mellitus. There is significant difference (p < 0.000) in serum sialic acid concentrations between at least two means, hence the null hypothesis is rejected. Serum sialic acid concentrations were significantly higher among the diabetic subjects (66.0 ± 11.7 mg %) as compared to the controls (55.2 ± 8.3 mg %), (p < 0.01). Significant higher values of serum sialic acid were found among diabetic nephropathy (71.6 ± 23.6 mg %) and retinopathy (77.9 ± 29.0) subjects as compared to the controls (P < 0.000) (Figure 1). The waist/hip ratio of the diabetic (0.92 ± 0.07), diabetic nephropathy (0.94 ± 0.06) and diabetic retinopathy (0.93 ± 0.09) subjects were significantly higher than the controls (0.86 ± 0.06) (P < 0.000). The systolic blood pressure was significantly higher in diabetic (144.4 ± 20.5 mmHg), diabetic nephropathy (160.3 ± 28.0 mmHg) and diabetic retinopathy subjects (162.8 ± 25.60) when compared to the controls (123.9 ± 14.5 mmHg) (p < 0.000). Similarly, triglyceride concentrations were significantly higher among diabetics (155.4 ± 114.8 mg/dl), diabetic nephropathy (149.4 ± 75.8) and diabetic retinopathy (176.5 ± 93.2) subjects as compared to controls (105.7 ± 65.1 mg/dl), however the diabetics with nephropathy showed moderate increase in triglycerides when compared to the controls. There was not much difference in the LDL values among the groups. The figure 2 explains the lipid profile status among the four study groups. It was found that there were significant increases in fasting blood glucose, urinary albumin and HbA1c among diabetics (p < 0.05). There was no significant change in the concentrations of C reactive protein, urinary sialic acid, HDL, VLDL, cholesterol and BMI between the four study groups. Serum sialic acid was significantly correlated with urine microalbumin (r = 0.484, p < 0.01), waist/hip ratio(r = 0.226, P < 0.05) and systolic blood pressure(r = .263, P < 0.01) Figures 3 and 4 represents the correlation of sialic acid with waist/hip ratio and systolic blood pressure respectively

**Table 1:** Serum inflammatory markers, metabolic and anthropometric variables in Caribbean Type 2 diabetics with and without microvascular complications

Variable	Controls (Mean ± SD) N = 44	Diabetics (Mean ± SD) N = 44	Nephropathy (Mean ± SD) N = 44	Retinopathy (Mean ± SD) N = 30	P value
BMI (kg/m^2^)	27.5 ± 7.34	29.1 ± 9.5	27.1 ± 6.9	28.7 ± 4.2	NS
Waist/hip ratio	0.86 ± 0.061	0.92 ± 0.07	0.94 ± 0.06	0.93 ± 0.094	0.000
Blood pressure (mmHg)	123.8 ± 14.5	144.4 ± 20.5	159.8 ± 6.92	162.8 ± 25.6	0.000
Serum Sialic acid (mg/dl)	55.2 ± 8.3	66.0 ± 11.7	71.6 ± 23.6	77.9 ± 29.0	0.01
Urine Sialic acid (mg/dl)	29.0 ± 15.1	38.5 ± 28.4	35.9 ± 38.9	55.1 ± 47.4	NS
Microalbumin (mg/l)	8.5 ± 12.5	27.9 ± 30.5	794.3 ± 805.9	351.1 ± 559.9	0.05
CReactive protein (mg/l)	2.0 ± 2.0	3.1 ± 4.2	3.5 ± 6.4	3.45 ± 3.8	NS
Cholesterol (mg/dl)	206.7 ± 53.7	189.7 ± 42.2	185.8 ± 61.4	216.0 ± 63.1	NS
Triglycerides (mg/dl)	105.6 ± 65.1	155.4 ± 114.8	149.3 ± 75.8	176.5 ± 93.2	0.01
HDL (mg/dl)	43.8 ± 10.5	44.3 ± 9.1	43.9 ± 10.2	44.8 ± 11.5	NS
LDL (mg/dl)	140.4 ± 53.5	114.3 ± 42.6	112.4 ± 56.3	135.9 ± 63.2	NS
VLDL (mg/dl)	21.6 ± 13.6	31.9 ± 22.5	30.1 ± 15.2	35.2 ± 18.6	NS
Glucose (mg/dl)	88.8 ± 9.7	135.5 ± 22.9	152.9 ± 74.9	157.7 ± 73.73	0.05
HbA_1_c (%)	4.3 ± 3.7	6.4 ± 2.0	7.7 ± 2.8	8.0 ± 2.7	0.05

P < 0.05 considered significant, NS = not significant.

## Discussion

Within recent years, there has been considerable interest and research into sialic acid and its use as a potential inflammatory marker for diabetes mellitus [11]. Previous reports have also indicated that serum sialic acid (SSA) concentrations were associated with an increased risk of cardiovascular disease in the diabetic population as well as the presence of micro-vascular diabetes related complications [12]. Our study primarily focuses on the relationship between sialic acid and metabolic variables. We also wished to investigate the relationship between sialic acid and lipid profiles and to determine whether sialic acid concentrations were increased in patients with diabetes with and without microvascular complications. Previous studies have indicated that SSA concentrations are elevated in diabetics (both type 1 and type2) with and without complications, while others have reported no such correlation [13,14]. Studies have also found that the presence or absence of this trend may be related to ethnicity [15].

Our research indicates that there is a significant increase (p < 0.01) in SSA concentrations in diabetic patients when compared to the control. This finding was also observed when patients with diabetic nephropathy and retinopathy were compared to the controls. It was noted that there was significant increase in SSA when diabetic group compared to diabetic retinopathy patients.

Serum sialic acid is a protein bound carbohydrate and occurs in combination with monosaccharides like galactose and mannose. Ninety percent is bound and almost none are free [16]. In serum they are generally bound to acute phase proteins [17]. There are several possible explanations for the increase in SSA concentrations. Several research studies have shown that the concentration of sialic acid in serum is elevated in pathological states when there is damage to tissue, tissue proliferation and inflammation [16]. Research studies have also indicated that vascular permeability is regulated by sialic acid moieties. The vascular endothelium carries a high concentration of sialic acid and hence extensive microvascular damage associated with non-insulin dependant diabetes mellitus (NIDDM), could account for its shedding into the circulation. This leads to an increase in vascular permeability and overall increased SSA concentrations [6,12].

Tissue injury caused by diabetic vascular complications stimulates local cytokine secretions from cells involved in the complications such as macrophages and endothelium. This induces an acute phase response which involves the release of acute phase glycoproteins with sialic acid from the liver into the general circulation again leading to increased SSA concentrations [11]. Another plausible explanation for the increases SSA is that there may be a difference in the ratio between the two forms of erythrocyte sialidases which are important in maintaining the viability of the erythrocyte and its survival in the circulating blood [18].

As expected the difference between SSA concentrations between diabetes with complications and healthy subjects were statistically more significant (p < 0.000) than the difference between diabetics and healthy subjects (p < 0.010). In diabetic nephropathy and retinopathy there is a further increase in micro vascular damage which may have resulted in the greater increase in the SSA concentrations observed.

The increase in urine albumin seen in diabetics as compared to the controls can be interpreted as an early sign of nephrogenic changes in those individuals. The increase in urine albumin was seen with diabetic nephropathy was significantly higher than the other group subjects. This can be attributed to the degradation of the glomerular basement membranes as well as the increased hypertension which are key characteristics of diabetic nephropathy [19].

Microalbuminuria, the dominant feature of diabetic nephropathy is defined as an albumin excretion rate of 20–300 mg/24 hrs. The presence of microalbuminuria is a marker of endothelial dysfunction whether or not it progresses. This marker indicates an increased risk of generalized atherosclerosis and increased mortality from cardiovascular disease [20].

With regards to diabetes-related complications, a positive correlation was established between urine albumin levels and serum sialic acid using the Pearson's Correlation Test. This association was also seen in earlier studies [21]. The actual cause of this occurrence is not known however several researchers have proposed a variety of mechanisms. One such mechanism includes the shedding of sialic acid into the circulation as a result of vascular endothelial damage. This result in the increased sialic acid as previously described. Vascular damage is seen throughout the body including the kidneys especially in diabetic nephropathy. As a result, there is increased filtration of albumin via the damaged glomeruli and hence an increased albumin loss in the urine [22].

With respect to the lipid profile the following observations were made. The cholesterol and LDL levels were unusually high in patients who were controls. This may be attributed to the unrestricted diets of these patients as well as possible inactive lifestyles [23]. The unexpectedly lower LDL levels in the diabetics with and without nephropathy may be attributed to a low fat and carbohydrate diet [24]. There was a significant increase in triglyceride levels when diabetics with and without microvascular complications compared with controls. An increased triglyceride level is a common feature of diabetes mellitus. Research has suggested that this is a result of reduced action of insulin on adipocytes resulting in suppression of lipolysis. This results in reduced hydrolysis of stored triglycerides and so a greater increase in non esterified fatty acids [25].

Increased triglyceride and LDL levels are risk factors for cardiovascular disease [26]. There was an increased TG level but not the expected simultaneous increase in LDL levels amongst the four groups as explained above. Another finding of our study was that serum sialic acid was not related to cholesterol, triglyceride, LDL and HDL levels.

There was a positive association between SSA and systolic blood pressure and as stated before there was also a positive correlation between SSA and urine albumin excretion. Blood pressure and microalbuminuria are also risk factors for cardiovascular disease [27]. Therefore it can be concluded that sialic acid may be considered as a possible marker for cardiovascular disease. Previous studies showed similar results [28,29]. There was a positive correlation between waist to hip ratio and SSA however this relationship was not found with BMI. This indicates that central adiposity may be an important marker of NIDDM as opposed to general obesity since waist to hip ratio is a specific indicator of central adiposity [30]. Central adiposity is a cardiovascular risk factor, hence these patients may have underlying microvascular complications and this may explain the correlation observed with SSA [31]. This further supports the theory that SSA can be used as a marker for cardiovascular risk.

In addition to sialic acid another inflammatory marker was assessed, C- reactive protein (CRP). There was an insignificant increase in the CRP levels amongst the four groups. This could be explained by the inherent inflammatory state in diabetics with and without complications [32]. There was no correlation between sialic acid and CRP suggesting that these inflammatory markers occur independently of each other.

In terms of limitations of this study the sample size could have been larger. Several patients were not certain about the type of medications that they were on and some of the patients were on antihypertensive drugs. The effects of these medications are unknown. Patients with diabetic nephropathy could also have had early signs of other complications such as retinopathy and neuropathy. There was no definite way of determining this and no way of assessing the impact of this on our study.

In the interest of future researchers who wish to conduct similar studies we have several recommendations that we wish to make: A larger sample size should be observed over a longer time period, multiple blood and urine samples from the same patient should be collected and 24 hr.urine samples should be collected instead of one time urine samples. Finally, we recommend that researchers take into account other diabetic complications such as diabetic retinopathy and diabetic neuropathy to allow for a more complete study.

## Conclusion

From this study several conclusions can be drawn. These include:(i) serum sialic acid concentrations were increased in type 2 diabetics with and without complications in the residents of Caribbean Island, Trinidad aged 15–60 (ii) serum sialic acid and microalobumin were positively correlated with hypertension and increased waist to hip ratio and therefore this suggests the potential use of serum sialic acid and microalbumin as a marker for cardiovascular risk (iii) serum sialic acid shows no correlation with components of the lipid profile (iv) waist to hip ratio may be a better marker of adiposity than BMI.

## Competing interests

The author(s) declare that they have no competing interests.

## Authors' contributions

SN was responsible for designing and carrying out the experiments

RL was responsible for providing the patients
